# Differential dopamine release by psychosis-generating and non-psychosis-generating addictive substances in the nucleus accumbens and dorsomedial striatum

**DOI:** 10.1038/s41398-021-01589-z

**Published:** 2021-09-13

**Authors:** Klara Danielsson, Rosita Stomberg, Louise Adermark, Mia Ericson, Bo Söderpalm

**Affiliations:** 1 Addiction Biology Unit, Department of Psychiatry and Neurochemistry, Institute of Neuroscience and Physiology, Sahlgrenska Academy, University of Gothenburg, Gothenburg, Sweden; 2 Department of Pharmacology, Institute of Neuroscience and Physiology, Sahlgrenska Academy, University of Gothenburg, Gothenburg, Sweden; 3 Beroendekliniken, Sahlgrenska University Hospital, Gothenburg, Sweden

**Keywords:** Physiology, Schizophrenia, Molecular neuroscience

## Abstract

Schizophrenia is associated with three main categories of symptoms; positive, negative and cognitive. Of these, only the positive symptoms respond well to treatment with antipsychotics. Due to the lack of effect of antipsychotics on negative symptoms, it has been suggested that while the positive symptoms are related to a hyperdopaminergic state in associative striatum, the negative symptoms may be a result of a reduced dopamine (DA) activity in the nucleus accumbens (nAc). Drug abuse is common in schizophrenia, supposedly alleviating negative symptomatology. Some, but not all, drugs aggravate psychosis, tentatively due to differential effects on DA activity in striatal regions. Here this hypothesis was tested in rats by using a double-probe microdialysis technique to simultaneously assess DA release in the nAc and associative striatum (dorsomedial striatum; DMS) following administration of the psychosis-generating substances amphetamine (0.5 mg/kg), cocaine (15 mg/kg) and Δ^9^-tetrahydrocannabinol (THC, 3 mg/kg), and the generally non-psychosis-generating substances ethanol (2.5 g/kg), nicotine (0.36 mg/kg) and morphine (5 mg/kg). The data show that amphetamine and cocaine produce identical DA elevations both in the nAc and DMS, whereas nicotine increases DA in nAc only. Ethanol and morphine both increased DMS DA, but weaker and in a qualitatively different way than in nAc, suggesting that the manner in which DA is increased might be important to the triggering of psychosis. THC elevated DA in neither region, indicating that the pro-psychotic effects of THC are not related to DA release. We conclude that psychosis-generating substances affect striatal DA release differently than non-psychosis-generating substances.

## Introduction

Schizophrenia is a neuropsychiatric disorder that affects ~0.3–0.7% of the global population, and for the afflicted individuals, the condition can cause life-long suffering and disability [1, 2]. Due to the chronic and debilitating nature of the disorder, the effects on society are marked, with WHO ranking schizophrenia as number 8 on the listing of disability-adjusted life years in age group 15–44 years [3, 4].

The symptomatology of schizophrenia is divided into three main categories—positive, negative and cognitive symptoms. The positive symptoms include hallucinations and delusions, and generally respond well to treatment with antipsychotics. Negative symptoms include a lack of motivation and energy, social withdrawal and overall negative affect. The last category, the cognitive symptoms, are comprised of executive dysfunction, lack of attentiveness and deficits in working memory. The negative and cognitive symptoms are both largely resistant to treatment with traditional antipsychotics [5]. Amongst the available antipsychotic treatments, only the atypical antipsychotic clozapine reliably improves the negative and cognitive symptoms, albeit with the risk of producing several severe side effects. This, and the fact that not all patients respond to the treatment [6], makes it a less-than-ideal treatment to target negative and cognitive symptomatology. In light of these difficulties, and the severely negative impact these symptoms have on the daily life of the patients, it is important to increase the number of effective treatments for negative and cognitive symptoms.

Extensive research (for a comprehensive review, see Hunt et al. [7]) and clinical experience indicate considerable comorbidity between schizophrenia and substance use disorders, e.g., nicotine use disorder, alcohol use disorder and cannabis use disorder, and also suggest that a not insignificant number of patients with schizophrenia use central stimulants (CS), such as amphetamine and cocaine, despite the fact that these substances are strongly pro-psychotic [8–10]. When asked, many patients report similar reasons; the CS enable them to get out of bed, take on the day, socialise and so on, suggesting that the use of CS is a way for the patient to self-medicate for the negative symptoms, albeit at the expense of aggravated positive symptoms. Drugs of abuse have been reported to increase DA primarily in the nucleus accumbens (nAc, ventral striatum), an important part of the brain reward circuitry, linked to the rewarding and reinforcing properties of drugs of abuse [11–13]. Furthermore, imaging studies have shown that the negative symptoms in schizophrenia are largely related to activity in the ventral rather than the dorsal striatum [14, 15], suggesting a possible link between low dopaminergic activity in ventral striatum and negative symptoms.

While most, if not all, major drugs of abuse increase DA release in the nAc, far from all of them produce or exacerbate positive symptoms. For example, opioids such as morphine and heroin readily increase accumbal DA levels in rodents [16], but are not known to produce psychosis. Likewise, nicotine robustly increases nAc DA, but does not worsen the positive symptoms. The reason for this is not entirely understood, but one possibility is that potentially psychosis-generating and non-psychosis-generating drugs differentially affect striatal DA.

Combining this knowledge with the translational aim of our research group, we were interested in using an animal model to investigate whether addictive substances that acutely may aggravate positive symptoms in schizophrenic patients or produce psychosis upon acute or sub-chronic use also in healthy individuals—psychosis-generating drugs (amphetamine, cocaine, THC)—have properties that differ from those of addictive substances that generally do not produce psychotic symptoms—non-psychosis-generating drugs (nicotine, ethanol, morphine). Dopamine, and more specifically DA D2 receptor activation, is of vital importance for eliciting psychosis, as suggested by the fact that in principle all antipsychotic medications possess DA D2 receptor antagonistic properties [17–19]. The connection between a hyperdopaminergic state and psychosis has since been strengthened by brain imaging studies showing that both the DA releasing effects of amphetamine as well as baseline DA levels are increased in patients with schizophrenia [20–23], most pronounced in the associative striatum [24]. Further, amphetamine-induced DA release in this area correlates with increased positive symptoms in schizophrenia [21–23]. Collectively, these findings suggest that the positive symptoms may be produced by a hyperdopaminergic state in the associative striatum.

In this study, we thus aimed to elucidate the tentative differential effects of psychosis-generating and non-psychosis-generating addictive drugs on nAc and dorsomedial striatum (DMS, the equivalent to associative striatum in rats) DA release, using a double-probe in vivo microdialysis technique. The present study serves as the first step towards the long-term goal to launch a thorough investigation into the potential benefits of targeted manipulation of nAc DA, utilising agents that exclusively elevate nAc DA without affecting DMS DA. Using a translational strategy, enabled by the close ties between clinical practice and experimental expertise in our research group, we hope to eventually be able to provide a novel approach to treat negative symptoms in schizophrenia.

## Methods

### Animals

Adult male Wistar Han rats (Envigo, Melderslo, Netherlands), weighing 280–350 g at the time of surgery (corresponding to ~9–10 weeks of age) were housed in groups of 3–4 per cage (55 × 35 × 20 cm) at constant room temperature (22 °C), relative humidity (65%), and reverse dark-light conditions. All animals had ad libitum access to standard rat feed and tap water. After arrival to the animal facilities, the animals were allowed to habituate to the environment for 1 week before surgery. This study was approved by the Ethics Committee for Animal Experiments, Gothenburg, Sweden and conducted according to national laws and guidelines for the care and use of laboratory animals.

### Experimental design

A total of 43 animals were included in the dataset, divided into the following groups; control (six animals receiving NaCl as vehicle, and two animals receiving a control solution for the THC experiment), nicotine (*n* = 6), amphetamine (*n* = 5), cocaine (*n* = 4), morphine (*n* = 5), ethanol (*n* = 7) and THC (*n* = 8). In a separate study, four animals received local administration of amphetamine to verify that the drug itself or the drug-elicited DA do not diffuse between the two areas, bringing the total number of animals to 46 in the two studies. Sample sizes for each treatment group were based on previous experience with the method. An additional five animals were excluded from analysis due to incorrect probe placement or excessive bleeding around the probe.

### Drugs

Nicotine (Sigma-Aldrich, Stockholm, Sweden), d-amphetamine (Apoteket AB, Stockholm, Sweden), cocaine (Apoteket AB), morphine (Apoteket AB) and ethanol (95%, KiiltoClean AB, Täby, Sweden) were dissolved in physiological saline solution (0.9% NaCl), pH-adjusted and administered s.c. or i.p. at a volume of 2 ml/kg or 5 ml/kg for EtOH. THC (Δ^9^-tetrahydrocannabinol solution, Sigma-Aldrich) was diluted in a 45% solution of β-cyclodextrin (Sigma-Aldrich) to a concentration of 1.5 mg/ml. The doses of ethanol (2.5 g/kg, i.p.) and nicotine (0.36 mg/kg, s.c.) were chosen based on the knowledge that these doses produce maximal and similar DA elevations in the nAc [25]. Since previous studies have demonstrated more pronounced DA elevations in ventral as compared to dorsal striatum following both these drugs [26] we hypothesised that these doses would produce maximal effects also in the DMS. The dose of morphine (5 mg/kg) was chosen in order to obtain nAc DA elevations in approximately the same range as following ethanol and nicotine and for the stimulants a moderately low dose of amphetamine (0.5 mg/kg) was chosen to be able to detect subtle differences in regional dopamine output whereas a moderately high dose of cocaine (15 mg/kg) was chosen. The THC dose (3 mg/kg) was chosen based on previous studies indicating that this dose may elevate DA in the nAc [27].

### Surgery

**Fig. 1:**
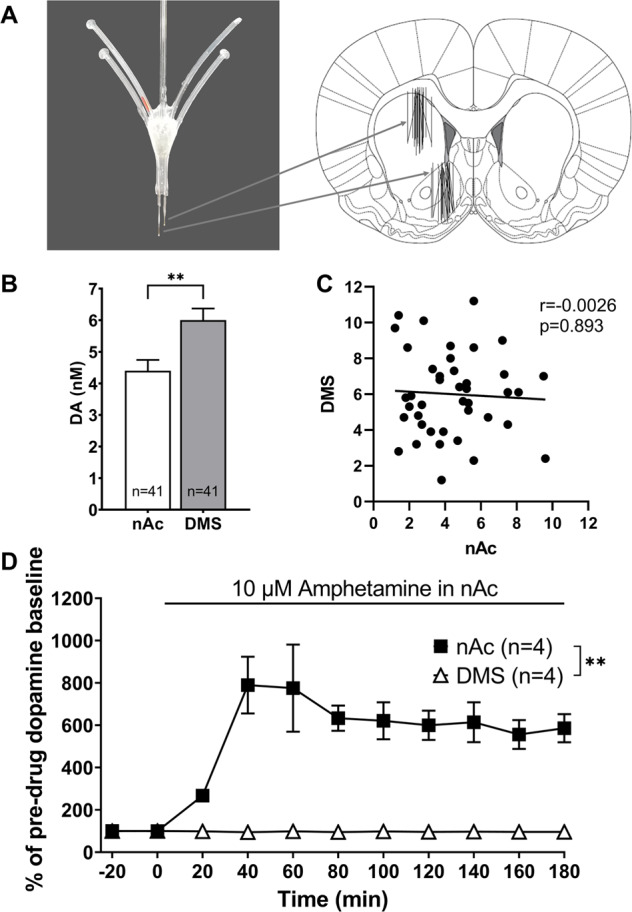
Higher dopamine dopamine levels in the DMS during basal conditions. **A** Photograph of the custom-made dual microdialysis probe used, with a schematic illustration of the probe placement in the DMS and the nAc. **B** Basal extracellular levels of DA in the DMS and nAc. **C** Correlation analysis of DA in the nAc and DMS, with no apparent correlation between basal levels of DA in the two regions. **D** Local administration of 10 µM amphetamine in the nAc elicits a substantial DA release in the nAc only. All values are presented as means ± SEM. *n* number of rats. **p < 0.005.

The in vivo microdialysis experiments were performed as previously described [28]. Animals were anaesthetised and equipped with a custom-made dual microdialysis probe (Fig. 1A), which permits simultaneous sampling from both the nAc and DMS. The dual probe, with an active space of 2 mm for each of the probes, was lowered into the nAc (A/P +1.85, M/L −1.4, D/V −7.8) and DMS (A/P +1.2, M/L −2.0, D/V −5.5 [29]). During the surgery, the animals received Marcain®, (buvipacaine, Apoteket AB) and Orudis® (2.5% ketprofen gel, Apoteket AB) as local analgesia and anti-inflammatory treatment, respectively. The animals were then placed in single housing cages and allowed to recover for ~48 h prior to the microdialysis experiment.

### In vivo microdialysis

On the day of the experiment, the animals were weighed (weight-loss exceeding 10% of pre-surgery weight were grounds for exclusion from the experiment (*n* = 0)) and then connected to a microperfusion pump (U-864 Syringe Pump, AngTho’s, Lidingö, Sweden) via a swivel, allowing the animals to move around the cage freely. During the entire experiment, the probes were perfused with Ringer solution at a rate of 2 µl/min, and dialysate samples were collected every 20 min. Once at least three consecutive stable DA values were obtained (±10%), pharmacological treatment was administered systemically, followed by a total of 180 min of sampling. In a separate experiment, 10 µM of amphetamine was dissolved in Ringer solution and perfused in the nAc probe only.

### Biochemical assay

Microdialysis samples were analysed for DA as previously described [30]. In brief, DA content was analysed by HPLC with electrochemical detection, using an external standard containing 3.25 fmol/µl of DA to identify the peak and quantify the content. The chromatograms were analysed using the Thermo Scientific Chromeleon Chromatography System (CDS) Chromeleon 7 (Waltham, Massachusetts, USA).

### Verification of probe placement

The animals were euthanized immediately following the experiment, their brains removed, fixed in formalin-free fixative (Accustain, Sigma-Aldrich) and stored at 4 °C. The brains were later sectioned and probe placement visually verified. Only animals with correct probe placement (Fig. 1A) and no signs of bleeding or extraneous damage to the tissue were included in the statistical analysis.

### Statistics

Statistical analysis of microdialysis data was carried out using a two-way analysis of variance with repeated measures. Basal levels of DA were analysed using a two-tailed *t*-test and Pearson’s correlation coefficient analysis. Values are expressed as mean ± SEM and a probability (*p*) value <0.05 was considered statistically significant. Normal distribution was tested using Sharpio–Wilk normality test. Statistical analyses were made using GraphPad Prism version 9.1.0 for Windows (GraphPad Software, San Diego, California, USA).

## Results

### Regional differences in dopamine levels during baseline conditions

When comparing basal extracellular levels of DA in the two brain regions, significantly higher levels were found in the DMS relative to the nAc (paired *t*-test: nAc vs. DMS, *t*_(40)_ = 3.10, *p* = 0.004, Fig. 1B), with no apparent correlation between the DA levels of the two regions (*r* = 0.054, *p* = 0.74, Fig. 1C). In order to assess the potential spread of drug or drug-induced DA between the two probes, amphetamine (10 µM) was perfused locally through the nAc probe. This elicited a significant DA release in the nAc, with no effect on DMS DA (nAc vs. DMS, brain region: *F*_(1, 6)_ = 31.1, *p* = 0.0014; time: *F*_(5, 30)_ = 7.09, *p* < 0.001; interaction: *F*_(5, 30)_ = 7.20, *p* < 0.001, Fig. 1D).

### Differential effects on striatal dopamine by psychosis-generating and non-psychosis-generating drugs

**Fig. 2:**
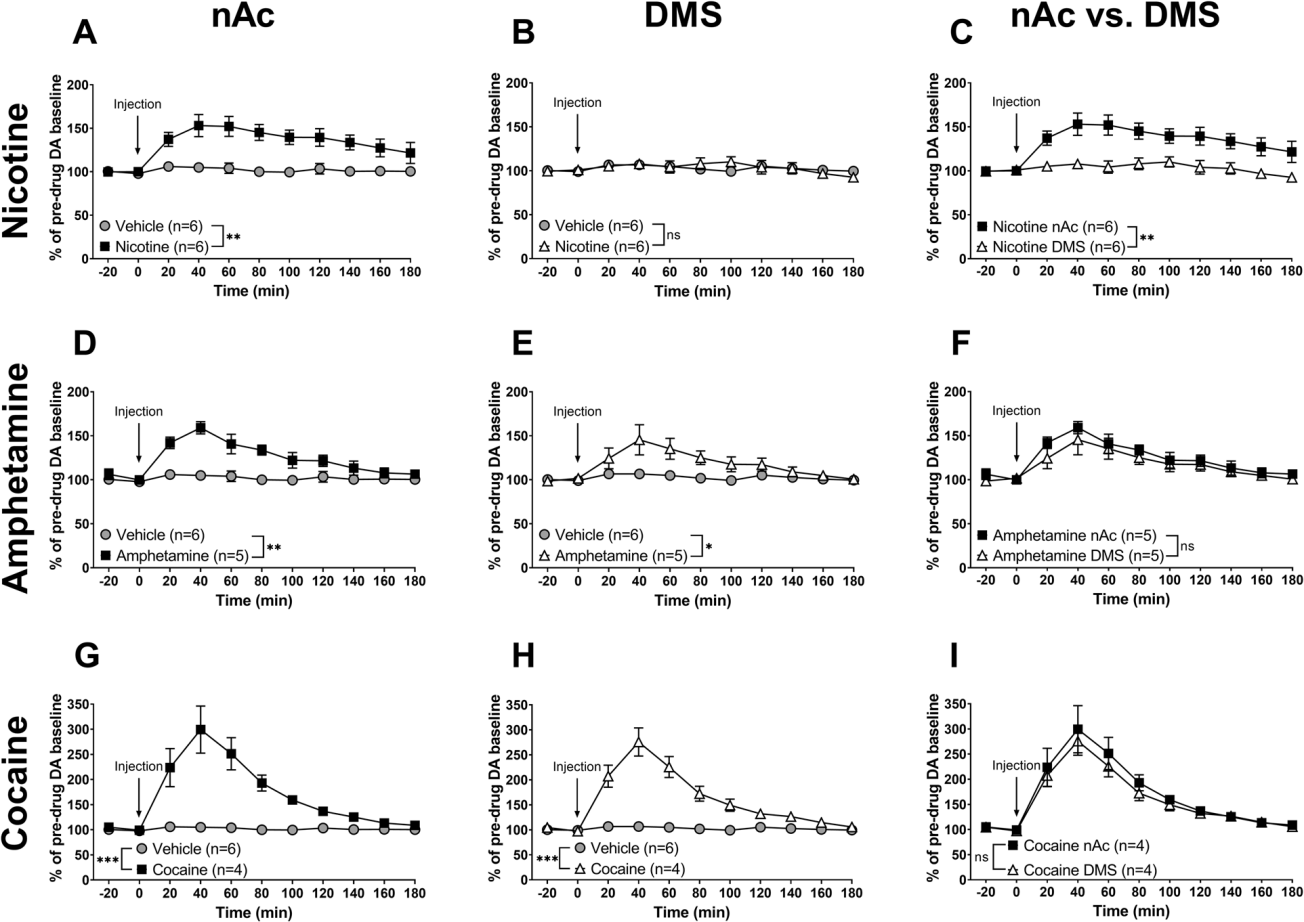
Differential DA release in the nAc and DMS by nicotine as compared to amphetamine and cocaine. **A**–**C** Systemic administration of nicotine (0.36 mg/kg s.c.) resulted in a significant elevation in extracellular DA in the nAc, but not in the DMS. **D**–**F** Amphetamine administration (0.5 mg/kg i.p.) produced a significant elevation of DA both in the nAc and in the DMS, with no significant difference between the two regions. **G**–**I** Cocaine (15 mg/kg i.p.) induced a robust DA elevation in the nAc and the DMS, with no significant difference in the DA response between the two regions. All values are presented as means ± SEM. *n* number of rats. *p < 0.05, **p < 0.005, ***p < 0.001.

Systemic administration of nicotine (0.36 mg/kg s.c.) produced a robust and rapid increase of nAc DA (nicotine vs. vehicle, treatment: *F*_(1, 10)_ = 17.2, *p* = 0.0020; time: *F*_(5, 50)_ = 2.10, *p* = 0.081; interaction: *F*_(5, 50)_ = 1.66, *p* = 0.16, Fig. 2A), but not DMS DA (nicotine vs. vehicle, treatment: *F*_(1, 10)_ = 0.188, *p* = 0.67; time: *F*_(5, 50)_ = 0.341, *p* = 0.89; interaction: *F*_(5, 50)_ = 1.91, *p* = 0.11, Fig. 2B), when compared to vehicle controls. A subsequent comparison of the DA-elevating properties of nicotine in the nAc and the DMS showed a significant difference between the two groups (nicotine—nAc vs. DMS: brain region: *F*_(1, 10)_ = 11.9, *p* = 0.0062; time: *F*_(5, 50)_ = 2.44, *p* = 0.047; interaction: *F*_(5, 50)_ = 2.45, *p* = 0.046, Fig. 2C).

Amphetamine (0.5 mg/kg i.p.) produced a significant elevation of DA both in the nAc (amphetamine vs. vehicle, treatment: *F*_(1, 9)_ = 36.1, *p* < 0.001; time: *F*_(5, 45)_ = 4.67, *p* = 0.016; interaction: *F*_(5, 45)_ = 2.94, *p* = 0.022, Fig. 2D) and the DMS (amphetamine vs. vehicle, treatment: *F*_(1, 9)_ = 8.15, *p* = 0.019; time: *F*_(5, 45)_ = 3.035, *p* = 0.019; interaction: *F*_(5, 45)_ = 1.76, *p* = 0.14, Fig. 2E) when compared to vehicle controls. Comparing the DA elevation elicited by amphetamine in the nAc with that in the DMS, there was no significant difference (amphetamine—nAc vs. DMS, brain region: *F*_(1, 8)_ = 0.797, *p* = 0.40; time: *F*_(5, 40)_ = 5.95; *p* < 0.001 interaction: *F*_(5, 40)_ = 0.300, *p* = 0.91, Fig. 2F).

Similarly, cocaine (15 mg/kg i.p.) produced a substantial increase of DA in both the nAc (cocaine vs. vehicle, treatment: *F*_(1, 8)_ = 33.3, *p* < 0.001; time: *F*_(5, 40)_ = 15.4, *p* < 0.001; interaction: *F*_(5, 40)_ = 14.0, *p* < 0.001, Fig. 2G) and DMS (cocaine vs. vehicle, treatment: *F*_(1, 8)_ = 86.1, *p* < 0.001; time: *F*_(5, 40)_ = 18.3, *p* < 0.001; interaction: *F*_(5, 40)_ = 16.1, *p* < 0.001, Fig. 2H) compared to control animals, with no significant difference in cocaine-induced DA elevation between the two regions (cocaine—nAc vs. DMS, brain region: *F*_(1, 6)_ = 0.416, *p* = 0.54; time: *F*_(5, 30)_ = 21.1, *p* < 0.001; interaction: *F*_(5, 30)_ = 0.109, *p* = 1.0, Fig. 2I).

**Fig. 3:**
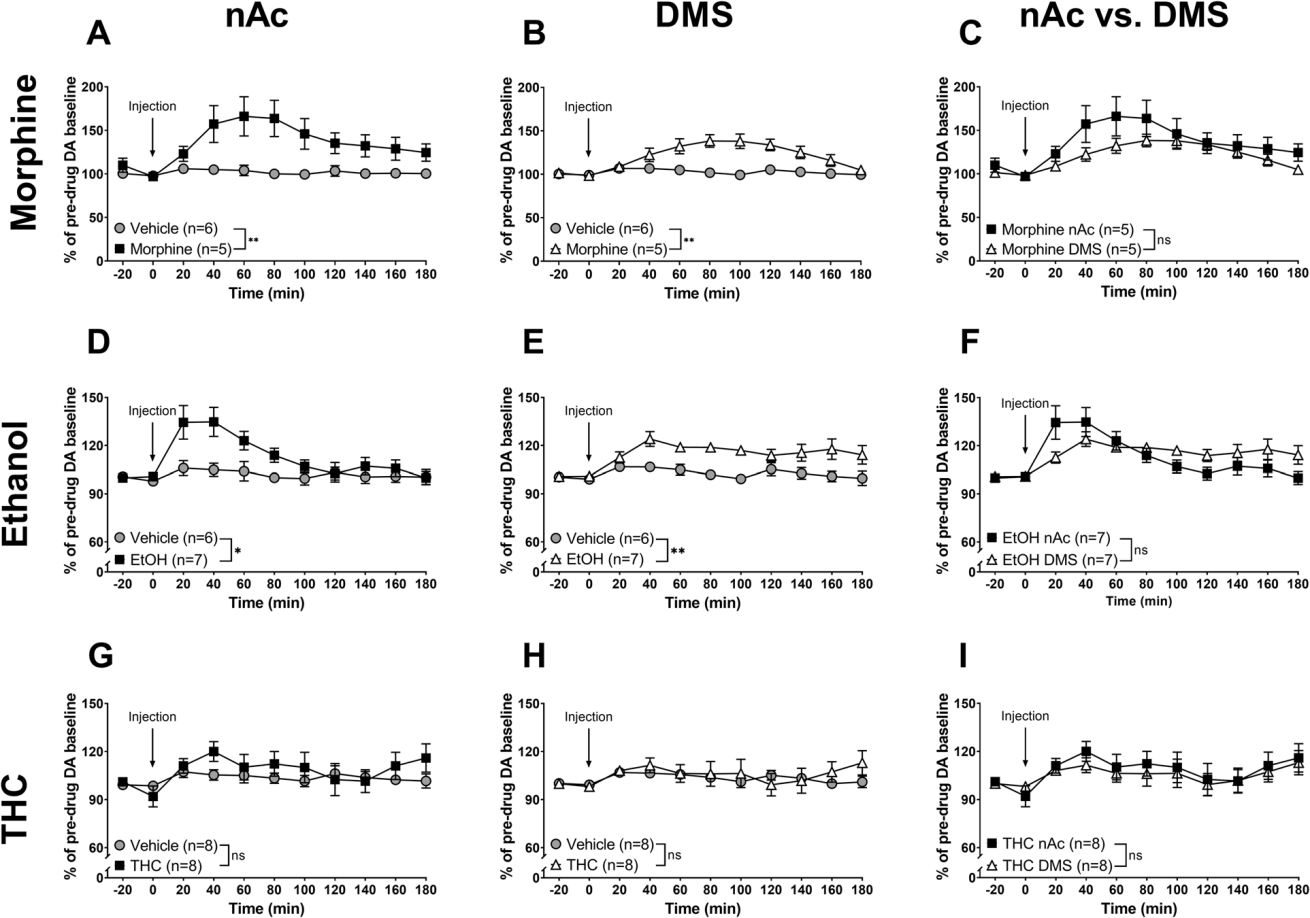
Qualitative differences in DA-elevating properties of ethanol and morphine in the DMS. **A**–**C** Systemic administration of morphine (5 mg/kg i.p.) produced an elevation in extracellular DA in both the nAc and the DMS, however, the effect differed qualitatively over time in the two regions. **D**–**F** Ethanol (2.5 g/kg i.p.) significantly increased extracellular DA levels in both the nAc and the DMS, an increase with a significantly different pattern in the two regions. **G**–**I** THC (3 mg/kg i.p.) failed to produce a significant increase in striatal DA, both in the nAc and the DMS. All values are presented as means ± SEM. *n* number of rats. *p < 0.05, **p < 0.01.

Systemic administration of morphine (5 mg/kg i.p.) produced a significant DA elevation compared to vehicle treated controls, both in the nAc (morphine vs. vehicle, treatment: *F*_(1, 9)_ = 8.62, *p* = 0.017; time: *F*_(5, 45)_ = 4.76, *p* = 0.0014; interaction: *F*_(5, 45)_ = 5.96, *p* < 0.001, Fig. 3A), and in the DMS (morphine vs. vehicle, treatment: *F*_(1, 9)_ = 26.11, *p* < 0.001; time: *F*_(5, 45)_ = 2.71, *p* = 0.032; interaction: *F*_(5, 45)_ = 5.79, *p* < 0.001, Fig. 3B). The morphine-induced DA response over time was qualitatively different in the two brain regions, as indicated by a significant interaction factor in the statistical evaluation (morphine nAc vs. DMS, brain region: *F*_(1, 8)_ = 1.27, *p* = 0.29; time: *F*_(5, 40)_ = 8.32, *p* < 0.001; interaction: *F*_(5, 40)_ = 2.45, *p* = 0.050, Fig. 3C).

Ethanol administration (2.5 g/kg i.p.) also produced a significant elevation of DA both in the nAc (ethanol vs. vehicle, treatment: *F*_(1, 11)_ = 6.89, *p* = 0.024; time: *F*_(5, 55)_ = 5.95, *p* < 0.001; interaction: *F*_(5, 55)_ = 3.43, *p* = 0.009, Fig. 3D) and in the DMS (ethanol vs. vehicle, treatment: *F*_(1, 11)_ = 15.8, *p* = 0.002; time: *F*_(5, 55)_ = 2.84, *p* = 0.024; interaction: *F*_(5, 55)_ = 2.79, *p* = 0.026, Fig. 3E). Ethanol, similar to morphine, increased DA in both regions in a qualitatively different manner (ethanol nAc vs. DMS, brain region: *F*_(1, 12)_ = 0.0931, *p* = 0.77; time: *F*_(5, 60)_ = 8.49, *p* < 0.001; interaction: *F*_(5, 60)_ = 5.87, *p* < 0.001, Fig. 3F).

For experiments involving THC, we needed to include a different vehicle solution (β-cyclodextrin with the addition of a small amount of ethanol) as this was used to dissolve THC. Following analysis of a few animals, no differences compared to saline treated rats were found with regards to DA, so the control groups were merged for analysis. Systemically administered THC (3 mg/kg i.p.) had no significant effect on striatal DA, compared to vehicle neither in the nAc (THC vs. vehicle, treatment: *F*_(1, 14)_ = 0.842, *p* = 0.37; time: *F*_(5, 70)_ = 0.872, *p* = 0.50; interaction: *F*_(5, 70)_ = 1.00, *p* = 0.42, Fig. 3G) nor in the DMS (THC vs. vehicle, treatment: *F*_(1, 14)_ = 0.0549, *p* = 0.82; time: *F*_(5, 70)_ = 1.57, *p* = 0.18; interaction: *F*_(5, 70)_ = 1.06, *p* = 0.39, Fig. 3H). Consequently, there was no difference in THC-induced DA output when comparing the two brain regions with each other (DA nAc vs. DMS, region: *F*_(1, 14)_ = 0.336, *p* = 0.57; time: *F*_(5, 70)_ = 2.17, *p* = 0.068; interaction: *F*_(5, 70)_ = 0.119, *p* = 0.99, Fig. 3I).

## Discussion

The main findings in the present study are that modest systemic doses of the potentially psychosis-generating drugs amphetamine and cocaine produced seemingly identical DA-elevating effects in nAc and DMS, whereas nicotine, which is a non-psychosis-generating drug, elevated DA in nAc only. Ethanol and morphine, which generally do not induce psychosis, increased DA in both regions, albeit qualitatively differently. The potential pro-psychotic drug THC failed to raise DA to any significant extent in both brain regions.

For the purpose of this study, a number of addictive substances were divided into psychosis-generating and non-psychosis-generating drugs. The basis for this division was the pharmacological potential or not of the substances to acutely aggravate psychosis in schizophrenic patients and to acutely, or after sub-chronic exposure, induce psychotic symptoms also in healthy individuals. Hence the division was not based on the association of substance use with the risk for developing schizophrenia, which is a more complex matter involving genetics as well as developmental and later environmental factors (e.g., exposure to cannabis, amphetamine or nicotine) [31–33].

In the present study, a double-probe approach was used, i.e., both regions were probed simultaneously in the same rat, reducing inter-individual variability. One concern with this method would be a possible spread of transmitter from one region to the other, especially considering the short distance between the two. However, baseline DA levels were significantly higher in the DMS and there was no correlation between the levels observed in the respective region. Further, reversed perfusion of high concentrations of amphetamine producing a pronounced DA elevation in the nAc did not alter DA content in the DMS. These results indicate that DA levels in the two regions are independent of each other and that even marked DA elevations in nAc are not detected by the probe in DMS.

There are multiple reports of DA elevations in nAc produced by systemic injections of nicotine, amphetamine, cocaine, ethanol and morphine in the rat, and the DA elevations reported are very similar to those found in the present study [34–38]. In comparison, there are considerably fewer reports on the effects of these drugs in the dorsal part of the striatum and we are aware of only one study (with ethanol) in which the effects specifically in the DMS have been studied [39]. In general, when comparing the effects on DA in the nAc and, presumably, the dorsolateral striatum (the exact location is often not stated in previous studies) the DA-elevating effects have been stronger in the nAc for all these drugs but also evident in the dorsolateral striatum. Here, we observed almost identical DA elevations in the DMS and nAc after moderate doses of amphetamine and cocaine. This thus differs from the contention that CS produce stronger DA effects in the nAc than in the dorsal striatum and suggests that there may be regional differences in this respect across the dorsal striatum. Further, nicotine elevated DA in the nAc only, i.e., this drug failed completely to produce an effect in DMS. This also differs from previous studies in the dorsal striatum and reinforces the notion that sub-regions of the dorsal striatum have to be considered. Finally, ethanol and morphine significantly elevated DA in both regions, albeit with slightly lower maximal effects and different time courses in the DMS, as judged from significant interaction terms. The DA elevations appeared slower in DMS as compared to nAc and hence there were qualitative differences in the DA response to ethanol and morphine in these two brain regions. The slower/lower DA response in DMS following systemic ethanol agrees with findings by Vena et al. [39].

There is a slight discrepancy in the reports on the ability of THC to produce reliable elevations of accumbal DA, with some suggesting that systemically administered THC is capable of elevating nAc DA [40], and others suggesting that it does not [41]. It also appears that differentiation of the shell vs. core regions of the nAc could be of importance [42]. Here, with the nAc probe placed in the shell/core border region (sampling from both regions), no statistically significant DA elevations were observed following THC, neither in the nAc nor in the DMS. Hence, based on these findings, it would appear unlikely that the pro-psychotic properties of THC are related to striatal elevations of extracellular DA.

The most interesting finding in the present study was the selective DA effect of nicotine in the nAc, whereas the more classical CS, amphetamine and cocaine, elevated DA in an almost identical manner in the two regions. This could potentially be explained by the fact that different DA neurons project to the two regions but that they all express DA reuptake carriers on their terminals. Thus, the CS, which produce their DA-elevating effect by blocking (cocaine) or reversing (amphetamine) the DA carriers [43, 44] produce similar effects in the two regions. Nicotine instead produces its DA-elevating effect in the nAc by stimulating nicotinic acetylcholine receptors (nAChRs) in the ventral tegmental area (VTA), probably located both on the DA neurons as such and on glutamatergic afferents to these neurons [45–47]. A plausible explanation to the lack of effect of nicotine on DA in DMS would then be that DA neurons projecting to this area, probably originating in the substantia nigra, have a different expression of nAChR than those projecting from the VTA [48].

The qualitative difference between the DA release produced in the nAc and DMS by ethanol and morphine could also be related to tentative differences in how these drugs interfere with different populations of DA neurons in the midbrain. Ethanol probably increases DA in the nAc by initially interfering with glycine receptors in nAc, which secondarily leads to indirect activation of specific sub-types of nAChRs in the anterior VTA [49, 50]. Morphine, on the other hand, is believed to produce its DA-elevating effect in nAc by stimulating mu-opioid receptors on GABAergic interneurons in VTA, thus reducing tonic inhibition of mesolimbic DA neurons [51]. The mechanisms by which these two drugs elevate DA in the DMS are unknown, but differences in the arrangement of receptor populations and/or with respect to which other neurons that are involved in the substantia nigra as compared to, e.g., the anterior VTA, may explain the qualitative differences here observed.

The working hypothesis for the present study was that psychosis-generating drugs of abuse and non-psychosis-generating drugs of abuse would differentially influence DA activity in nAc (reward area) and DMS (“psychosis” area). The profound difference between nicotine and the CS amphetamine and cocaine supports this hypothesis. Nicotine is commonly used by patients with schizophrenia, likely for pro-cognitive effects by elevating DA in frontal cortex [52] but probably also for alleviating negative symptoms by robustly elevating DA also in nAc, as observed here and in other studies [26, 53]. Despite a high prevalence of nicotine use, smoking in schizophrenic patients is not known to exacerbate psychosis, which is supported by the present finding that nicotine completely failed to alter DA levels in the DMS. The stimulants, in contrast, elevated DA in both regions, which fits with clinical experience and brain imaging studies that CS may improve negative symptomatology but at the cost of increased psychotic symptoms [21]. Unfortunately, schizophrenic patients are more sensitive than controls to the DA releasing effect of amphetamine, especially in the associative striatum [24], making it difficult to obtain relief of negative symptoms without exacerbating positive symptoms with this drug.

Even though long-term heavy ethanol intake eventually may result in alcohol hallucinosis [54], and psychotic features may appear upon alcohol withdrawal, these psychotic symptoms are usually distinct from those in psychotic disorders. Similarly, morphine or heroin generally do not produce psychosis. On the contrary, opiates have been suggested to produce antipsychotic effects [55, 56]. Therefore, ethanol and morphine are not psychosis-generating substances in the same manner as e.g., CS and THC. Interestingly, both ethanol and morphine produced slow and less pronounced DA elevations in DMS than in nAc, and not as pronounced elevations as the stimulants in the DMS. These qualitative and quantitative differences could possibly be the reason why these drugs most often are not psychosis-generating. For example, a slow rise in DA levels may allow DA D2 receptor desensitization before DA reaches top levels [57], a mechanism that would oppose the generation of psychotic symptoms. Furthermore, we know from previous studies that considerably larger striatal DA elevations can be obtained following higher doses of the stimulants, increasing the risk for psychosis, whereas higher doses of at least ethanol and nicotine are unlikely to produce larger DA-elevating effects [35, 39, 58]. However, as pointed out with respect to alcohol hallucinosis, factors related to sub-chronic and chronic use are also involved in pro-psychotic actions, e.g., sleep deprivation in the case of stimulants and possibly use pattern (binge vs. continuous intake). Whether such chronic effects are related to alterations of DA (e.g., sensitisation) and/or other systems and differ among and between psychosis-generating (amphetamine psychosis is e.g., more common than cocaine psychosis) and non-psychosis-generating addictive substances remain to be elucidated.

The present results in rats are in line with PET studies in humans showing DA release both in the ventral and dorsal striatum following CS and that release in the dorsal striatum is related to psychotic symptoms [20–24]. The ethanol results also agree with findings in humans of ethanol-induced DA release in the ventral striatum [59–63], whereas DA release has not been described in the associative striatum, suggesting that it is either absent or too small to detect with PET methodology. Further, in congruence with our results most PET studies report nicotine-induced DA release in the ventral striatum [64–68], whereas only a few studies have observed limited DA release in the associative striatum [69]. Opiates slightly release DA in ventral but not dorsal striatum in healthy volunteers (morphine) [70], but not in individuals with opioid dependence [71, 72]. Finally, THC produces no or very limited DA release in the human ventral striatum [73–75] but may cause some release in the caudate and cortical areas [74, 76]. Thus, also in humans, the non-psychosis-generating drugs ethanol, nicotine and morphine release DA in the ventral striatum but not to any significant extent in the associative striatum, whereas the psychosis-generating CS produce clear-cut DA elevations in both regions. THC, on the other hand, leaves ventral striatum DA largely untouched but may produce psychosis via DA in caudate putamen/temporal cortex or via mechanisms unrelated to DA. Taken together, these findings in rats and humans argue against the previously held notion that psychosis is related to DA in the ventral striatum and for that it is instead related to enhanced DA activity in the associative striatum/DMS (cf. also McCutcheon et al. [24]).

In conclusion, the results presented here suggest that strong CS with psychosis-generating properties, such as amphetamine and cocaine, elevate DA similarly in the nAc and the DMS. Drugs generally not considered psychosis-generating either do not elevate DA in the DMS or do so in a significantly different way with a slower rise than in the nAc. These findings lend credence to the tentative notion that associative striatum (DMS) DA plays a part in the expression of psychotic symptoms, whereas ventral striatum (nAc) DA may be non-psychotic and instead possibly beneficial for alleviating negative symptomatology.
